# Immunohistochemical Analysis of BRAF (V600E) Mutation and P16 Expression in Malignant Melanoma in Lagos, Nigeria: A 10-Year Retrospective Study

**DOI:** 10.1155/2019/1628247

**Published:** 2019-04-17

**Authors:** O. Obadofin, K. Badmos, N. Orsi, M. Bipin, O. Rotimi, A. Banjo

**Affiliations:** ^1^Department of Anatomic and Molecular Pathology, Lagos University Teaching Hospital, 100254 Lagos, Nigeria; ^2^Leeds Institute of Cancer & Pathology, Saint James's University Hospital, Leeds, UK; ^3^Department of Histopathology, Saint James's University Hospital, Leeds, UK

## Abstract

**Background:**

In Blacks, malignant melanoma (MM) is associated with greater morbidity and mortality compared to Caucasians. MMs with BRAF V600E mutation as well as those with loss of p16 protein expression are associated with aggressive behavior and worse prognosis.

**Objectives:**

We determined BRAF (V600E) mutation status and loss of p16 expression in MM cases in Lagos, Nigeria, and correlated these with histopathologic parameters and patients' age.

**Methods:**

Forty-five cases of MM received between January 2005 and December 2014 in the Anatomic and Molecular Pathology Department of Lagos University Teaching Hospital were subjected to immunohistochemical studies to determine BRAF V600E mutation and p16 protein expression. These included cutaneous (*n*=37), musosal (*n*=3), and ocular MM (*n*=2) as well as lymph node metastatases (*n*=3).

**Results:**

BRAF (V600E) mutations were detected in 5/45 (11%) while 31/45 (69%) of the cases had loss of p16 expression. No statistically significant association was found between the BRAF (V600E) mutation, loss of p16 expression, and histologic parameters such as histologic variant, Clark level, Breslow thickness, and ulceration.

**Conclusion:**

BRAF (V600E) mutation was detected only in a small proportion of cases while loss of p16 expression occurred in most cases which also had high Clark level, high Breslow thickness, and ulceration.

## 1. Introduction

Malignant melanoma (MM) is the most lethal of all cutaneous malignancies, accounting for 79% of skin cancer-related mortality [1]. It is one of the commonest cancers in Caucasians whose incidence has been increasing at an alarming rate [2, 3]. In Australia, it is referred to as the national cancer since the country has the highest incidence of MM globally, where it accounts for about 10% of the national cancer burden [4].

Previous studies have revealed that MM is the second commonest skin cancer in Nigeria, representing as much as 37.4% of histologically diagnosed skin cancers in some series [5–8]. Most patients present with late stage disease: in one study 69% of cases were of Clark level V, with as many as 95% of cases occurring on the foot [6].

Despite its aggressive nature, MM remains highly curable if detected early. Five-year survival is approximately 90% overall and exceeds 98% when disease is detected at the localized stage [9]. Survival is worse with increasing tumour size (>2cm), increasing depth of invasion (Clark level >II and Breslow thickness >2mm), ulceration, and presence of distant metastasis. With metastatic disease, 5-year survival is only 15%, and median survival is <1 year. At presentation, about 10% of melanomas have metastasized [9, 10].

Although the incidence of melanoma is much lower in Blacks than in Caucasians due to protection offered by higher skin melanin concentrations, the advanced stage of disease at presentation as a result of delayed diagnosis translates into greater morbidity and mortality [11, 12]. A study carried out in South Africa revealed that clinical stage at presentation was stage I: 32%, stage II: 52%, stage III: 14%, and stage IV: 2%. In 58% of the cases, tumour size was greater than 40mm in at least one dimension, and 43% of patients died within one year of presentation [13]. In another study in Tanzania, Clark's level I and II diseases were not seen and Clark's levels III, IV, and V accounted for 27.9%, 17%, and 26.9% of cases, respectively, while Clark's staging was not performed in the remaining cases [14].

The identification of some of the molecular changes that drive the carcinogenesis in melanoma has allowed the development of targeted therapy that can improve survival in advanced disease. These include activating mutations in the BRAF gene and loss of p16 protein expression [15, 16].

The BRAF gene located on chromosome 7q34 encodes a serine/threonine-protein kinase BRAF, which belongs to the family of growth signal transduction nonreceptor protein kinases [17]. Oncogenic BRAF activation leads to constitutive kinase activity and phosphorylation of downstream targets of the RAS/RAF/MAPK signaling pathway. Gain-of-function BRAF mutations have been identified in 7% of other human cancers, such as colorectal cancer, melanoma, papillary thyroid carcinoma, and some lymphomas [18].

The detection of BRAF mutations in melanomas, the development of inhibitors of the BRAF (V600E) protein, and the impressive clinical responses in a number of patients with advanced and metastatic disease treated with BRAF inhibitors have had a significant effect on the diagnostic work-up and management of patients with melanoma [16].

The gene CDKN2A is located on 9p21 and encodes three distinct proteins; p16 also known as INK4A (inhibitor of cyclin-dependent kinase 4), p15/INK4B, and p14/ARF (alternative reading frame). The p16 protein is responsible for controlling the G/S cell cycle transition. It prevents the phosphorylation of the retinoblastoma protein (pRB) by binding to CDK4 and CDK6 which inhibit the formation of the CDK4/6/cyclin D complex, responsible for phosphorylating the pRB [19].

Decreased expression of p16 (protein and mRNA expression) has been associated with clinical progression of melanoma in both familial and sporadic cases [20]. The loss of p16 in melanoma has been associated with increased tumour thickness and Clark's level, increased proliferation rate/ higher mitotic count, and risk of disease relapse and has been shown to be an independent prognostic indicator of decreased survival [21].

No study, however, has been carried out to determine the BRAF V600E mutation status and p16 expression in melanoma within the Nigerian population.

## 2. Materials and Methods

Paraffin embedded tissue blocks and haematoxylin and eosin (H&E) stained slides of forty-five (45) histologically diagnosed cases of MM seen over a period of 10 years at the Anatomic and Molecular Pathology Department of Lagos University Teaching Hospital (LUTH), Lagos, from January 2005 to December 2014 were retrieved from archives.

Relevant information such as age, hospital number, laboratory number, and clinical detail were extracted from the departmental cancer registry and from patient's folders.

Fresh sections from the tissue blocks were taken in situations where the original slides were not found or had been damaged.

Cutaneous MM cases were classified into histologic variants according to the 2006 WHO criteria [22]. The Clark's level, Breslow thickness, and ulceration status were also determined.

Immunohistochemical (IHC) studies to determine mutant BRAF V600E and p16 protein expression was done using the VE1 (Bioscience) and anti-CDKN2A/(Vector) monoclonal antibodies, respectively.

The VE1 antibody staining for BRAF (V600E) mutation was scored as positive when the majority of viable tumour cells showed brown cytoplasmic staining. Staining was scored as negative when there was no cytoplasmic staining or only isolated nuclear staining, weak staining of single interspersed cells, or staining of monocytes/macrophages. The positive control was a melanoma of known BRAF mutation status, while negative controls were normal keratinocytes.

Staining for p16 protein expression was evaluated as the percentage of positive tumour cells and subdivided into scores: Score 1/Negative, absent red nuclear staining of all <30% of the tumour cells show red nuclear staining; Score 2/Positive, ≥30% of the tumour cells showed red nuclear staining. The positive control was cervical cancer tissue while normal keratinocytes were used as negative control.

The data were analyzed using SPSS version 20 (IBM SPSS Statistics for Windows, Version 20.0 Armonk, NY: IBM Corp). Chi squared analysis was used to determine the relationship between mutant BRAF V600E expression/loss of p16 protein expression and patient age, histologic variant, Clark's level/stage, Breslow thickness, and ulceration. The alpha was set at 5% and p-value of less than 0.05 considered as statistically significant (Table 1).

## 3. Results

There were 42 primary melanomas which consisted of 37 cutaneous MM, 3 mucosal MM, and 2 ocular MM. There were 3 lymph node metastases.

The age range was from 24 to 85 years while the median age was 50 years.

Male to female ratio was 1: 1.5.

Cutaneous MM were predominantly located on the foot (68%, n=25/37). A minority were located on the leg (8%, n=3/37), trunk (8%, n=3/37), upper limb (5%, n=2/37), gluteus (5%, n=2/37), neck (3%, n=1/37), and scrotum (3%, n=1/37).

Histologically, the cutaneous MM cases were nodular MM 81%, 30/37, acral lentiginous MM 16%, 6/37, and desmoplastic MM 3%, 1/37.

Eighty-eight percent (33/37) of the cutaneous MM cases were Clark's stage IV and V. Eighty-four percent of the cutaneous MM cases (31/37) had Breslow thickness ≥4mm. Ulceration was present in 67% (25/37) cases.

**Figure 1:**
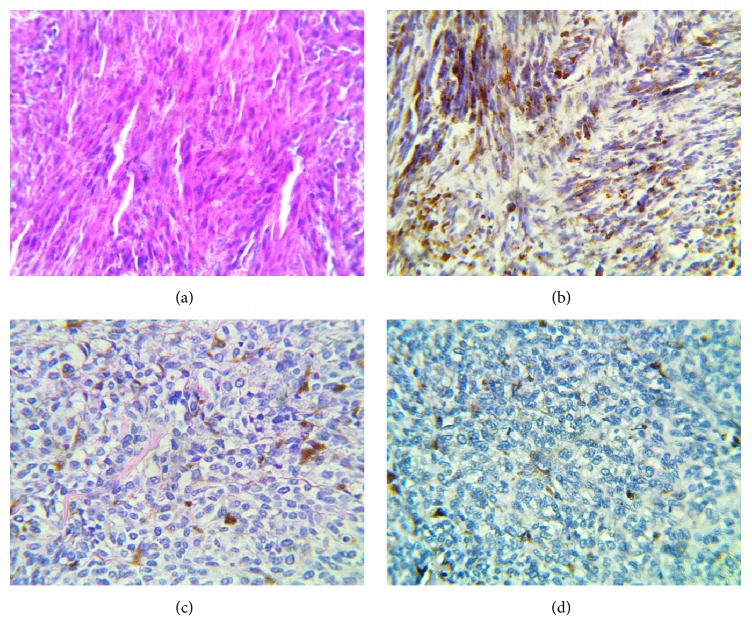
(a) Photomicrograph of case 44 stained with H&E (Magnification x 400); (b) positive staining with VE1 antibody for BRAF V600E in case 46 (Magnification x 400). Note the brown cytoplasmic staining of the tumour cells; (c) photomicrograph of case 25 stained with H&E (Magnification x 400). There are scattered melanin laden macrophages in the background; (d) negative staining with VE1 antibody for BRAF V600E in case 25 (Magnification x 400). There is no brown cytoplasmic staining of the tumour cells.

BRAF (V600E) mutation was detected in 5/45 (11%) which included 4 cutaneous MM and 1 lymph node metastasis (Figure 1).

**Figure 2:**
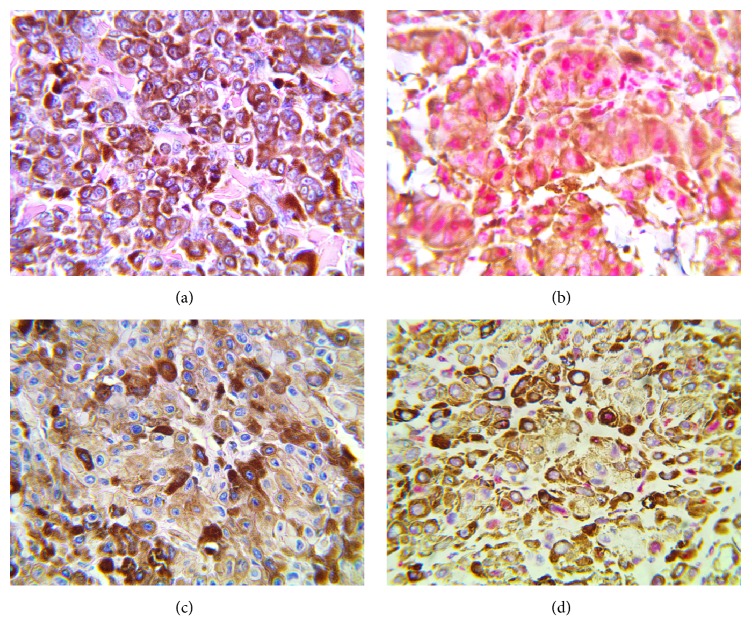
(a) Photomicrograph of case 17 stained with H&E (Magnification x 400). The tumour cells are heavily pigmented; (b) photomicrograph showing red nuclear staining of case 17 by the anti-CDKN2A/p16INK4a antibody (Magnification X 400); (c) photomicrograph of case 12 stained with H&E (Magnification x 400). The tumour cells are heavily pigmented; (d) photomicrograph showing no nuclear staining by the anti-CDKN2A/p16INK4a antibody (magnification X 400).

Loss of p16 expression was detected in 69% (31/45) of the cases consisting of 27 cutaneous MM, 2 mucosal MM, and 2 lymph node metastatases (Figure 2).

## 4. Discussion

A substantial proportion (86%) of the cases in this study were of Clark's levels IV and V similar to 88% reported in Jos by Mohammed et al. [8] There were no Clark's level I and II cases in this study. Literature from other parts of Nigeria, Africa, and in African-Americans showed similar findings [14]. A probable attribute could be late presentation, as most of these tumours occur on the foot without notice until they subsequently ulcerate. Such patients would have been managed for chronic foot ulcers over a period of time before the eventual diagnosis of melanoma. This emphasizes the need for increased patient and physician education in recognizing melanoma early in other to forestall these findings.

Moreover, an overwhelming 84% (36/43) of the cases had Breslow thickness ≥4mm in contrast to existing studies in Caucasians where much thinner lesions are typically encountered [2]. The implication of late presentation of patients with melanoma is that the disease becomes clinically advanced and aggressive with lymph node metastases. In a series in Cameroon, 32% of the patients that presented with advanced disease were said to have died within 12 months of presentation [12].

BRAF (V600E) mutation was detected in only 11% of the MM cases in this series. This is far less than that reported in Caucasians, where the incidence of this mutation is greater than 40% [18, 23]. While this may suggest that most of the MM in the present study are wild type, these findings could also reflect the fact that the BRAF mutation harboured by some of the cases may be among the minor ones such as BRAF (V600K), BRAF (V600R), BRAF (V600 ‘E2'), and BRAF (V600D) [24]. It is also of note that the Caucasians that record high percentage of BRAF mutation actually present early and with thinner lesions. This suggests that late presentation is actually an important factor in making melanoma in Blacks more aggressive and almost a death penalty.

Regardless, BRAF kinase inhibitors would be effective in treating only very few of our patients.

Loss of p16 protein expression was seen in the majority (69%) of the MM cases in this study. This is comparable to findings in Caucasians by Parvey et al. in which 52% of cases showed loss of p16 expression [25]. Straume and coworkers also reported that 45% and 77% of the primary and metastatic melanoma cases showed no nuclear staining, respectively [26]. This suggests that the CDKN2A gene may be mutated in the majority of our cases resulting in loss of p16/INK4a expression. This may probably explain the increased tumour thickness and Clark's level that were recorded in this study and which have been documented in studies carried out on Caucasians [26].

Nodular melanoma is the commonest type of melanoma in Blacks and it occurs usually on the lower limbs [22]. Our study agrees with this submission with 81% of our cutaneous MM cases being nodular melanoma histologically and 75% of the cutaneous MM cases occur in the lower limbs (leg and foot).

There was a trend for a higher percentage of nodular melanoma cases (88%) to have a loss of p16 expression in comparison to acral lentiginous (50%) (*P*=0.064). This compares with a study by Parvey et al. who reported nodular melanoma to have significantly lower levels of p16 expression than its superficial spreading counterpart [25].

In the present study, a higher proportion of Clark's stage IV and V cases had loss of p16 expression. This is also in agreement with the study by Parvey et al. who showed that loss of p16 staining was associated with thicker lesions. This supports the notion that the loss of p16 is a late event in the progression of sporadic primary melanoma and its association with more aggressive tumour [25].

## 5. Conclusion

Loss of P16 expression was seen in the majority of the cases of MM in LUTH; these cases also had high Clark's stage, increased Breslow thickness, and showed ulceration. BRAF (V600E) mutation was detected only in a small proportion of MM cases. Majority, over 80%, of the cases presented at advanced stage making this a critical point for intervention by increased and continuous patient and physician education in recognizing early signs of melanoma.

## Figures and Tables

**Table 1:** Correlation between P16 expression and prognostic factors.

Variables	P16		*p *value
	POSITIVE *n*=14(%)	NEGATIVE *n*=31(%)	
*Age group (years)*			
<40	2 (40)	3 (60)	0.123
40-49	7 (54)	6 (46)	
50-59	1 (14)	7 (86)	
60-69	4(33)	8 (67)	
≥70	0(0)	7(100)	
*Clark's Stage*			
I	0(0)	0(0)	0.627
II	0(0)	0(0)	
III	2(40)	3(60)	
IV	6(25)	18(75)	
V	5(45)	8(55)	
*Breslow thickness*			
<4mm	1(14)	6(86)	0.405
≥4mm	12 (36)	23(64)	
*Ulceration*			
Absent	4(25)	12(75)	0.282
Present	9(35)	17(65)	

*Variant (Cutaneous MM)*	*n*=10	*n*=27	
Acral lentiginous	2(50)	2(50)	0.064
Desmoplastic	1(100)	0(0)	
Nodular	7(22)	25(88)	

*∗*1 lymph node was P16 positive, while 2 were P16 negative. Histologic variant applies only to cutaneous MM, n = 37.

## Data Availability

The data used to support the findings of this study were supplied by Department of Anatomic and Molecular Pathology, Lagos University Teaching Hospital, Nigeria. Requests for access to these data should be made to Dr. Omobolade Obadofin, boladeoshaks@gmail.com.
